# Susruta and Wise on Ancient Indian Medicine

**Published:** 1847-04

**Authors:** 


### Art. XVII.

1. *The Susruta, or System of Medicine taught by Dhanwantari, and composed by his Disciple, Susruta (in Sanscrit).* Edited by Sri Madusu dana Gupta, Teacher of Medicine in the Sanscrit College; and printed by order of the Education Committee, at the Education Press.—*Calcutta,* English era 1825, Saka 1757. 8vo, pp. 378.
2. *Súsrutas. Ayurvêdas, id est Medicinæ Systema, a venerabili D'hanvantare demonstratum, a Súsruta Discipulo compositum.* Nunc primum ex Sanskrita in Latinum sermonem vertit, introductionem, annotationes, et rerum indicem adjecit Dr. Franciscus Hessler.—*Erlangæ,* 1844. First Part, 8vo, pp. 206.
3. *Commentary on the Hindu System of Medicine.* By T. A. Wise, m.d., Bengal Medical Service.—*Calcutta,* 1845. 8vo, pp. 431.

Distinguished as is the present age for discoveries in science, as well as for their application to practical purposes, it is not less so for investigations respecting the manners and customs, the language and history of the civilised nations of antiquity. Among these may be instanced, the knowledge which we have attained of the hieroglyphical writings of the Egyptians, and consequently of their chronology and history, in consequence of the discoveries first made by a medical philosopher, Dr. Young, and since continued by Champollion and Leipsius, as recorded in the works of Sir G. Wilkinson, of the Chevalier Bunsen, and of others. These researches have necessarily elucidated many subjects referred to in the Bible, while its geography and natural history have in many instances been clearly made out by the remarkable resemblance, which still exists between the present Arabic names of places and of things, to those which are made use of in the ancient, but cognate Hebrew. Of this numerous instances are given in the Travels of Dr. Robinson, and in Dr. Kitto's 'Cyclopædia of Biblical Literature.' The discoveries and works of Sir Charles Fellows have clearly shown, that many of the arts in which the Greeks attained eminence, were known to and introduced into Lycia, by a still more ancient people from some eastern country. So the recent excavations in the neighbourhood of Mosul and of Bagdad, into what appeared to be only mounds of earth and heaps of stone, have revealed numerous chambers and extensive halls of palaces, having their walls of marble covered with numerous and long inscriptions, and with sculpture, which is described as original in design and finished in execution, and displaying a knowledge of the anatomy of the human frame. Some of these are stated to afford a complete history of the military art amongst the Assyrians, and prove their intimate knowledge of many of the machines of war, the invention of which has been attributed to the Greeks and Romans, such as the battering-ram, the tower moving on wheels, the catapult, &c. (Athenæum, 1846, p. 1047.) So the cuneiform inscriptions of Persia, which so long resisted the corroding effects of time and all the efforts of the learned, have at length been translated by Major Rawlinson (Journ. Asiatic Soc., Sept. 1846), and found to be chiefly of the age of Cyrus the Great, of Darius, and of his son Xerxes, and in a language which bears a strong affinity to the Sanscrit of the Vedas, "but is at the same time distinguished from it by that uniform permutation, both of alphabetical powers and of grammatical inflexions, which points to a very remote period for their common separation from the parent stock." These discoveries Major Rawlinson believes are but a prelude to others of far greater moment, alluding probably to the Median and Babylonian inscriptions. If we look still further east, we find that, long as Sanscrit has been studied, it is only within the last fifteen years that the inscriptions cut into the solid rock or engraved on metallic pillars have been deciphered by the late lamented James Prinsep. These are in the ancient Pali language, of which the old Sanscrit is the type. History is much interested in this discovery, for the deciphering of innumerable coins of ancient Bactria, commencing with the third of the Seleucidæ and his known successors, and continuing for many centuries, has revealed a series of unrecorded Bactrian princes, and given some dates to the loose and inaccurate chronology of India. But the history of medicine is also much interested in this question, as one of the earliest of the inscriptions, and which bears indubitable proofs of the third century before the Christian era, is a *medical edict,* promulgated through the provinces of north-western India.

The history of medicine is therefore not unconnected with these investigations. That medicine was early practised by the Egyptians, Assyrians, and Persians is well known, and will be readily admitted when people become acquainted with the high perfection which many of the arts of life had attained among these early civilized nations. We know also that the nations of the East had early established for themselves so high a character for knowledge and wisdom, as to have been visited for the purposes of instruction, and during a series of ages, by the sages of Greece, as by Democritus, Pythagoras, and others. These on their return home established schools, from which new systems of philosophy and discoveries in science were promulgated. Information on the subject of medicine is so extremely scanty, as to give rise to doubts whether it could have made any greater progress than may be observed among some rude and uncultivated nations. Medicine is, however, considered by some to have originated in Egypt, and at all events that the Greeks obtained their first knowledge of it from that country; and, as Herodotus mentions that certain diseases were treated by particular persons, the fact would indicate that some division, and necessarily some progress, had already been made in the profession. They were said also to have possessed a kind of Egyptian encyclopædia of medicine in the books of Hermes, as these are stated to have treated—1, of anatomy; 2, diseases; 3, instruments; 4, remedies; 5, diseases of the eye; and 6, of the diseases of women. Whether this be authentic or not, there can be no doubt that Egypt was considered fertile in drugs in the time of Homer; and we have continued to talk even to our own times of Thebaic preparations, and of one of copper as "mel Ægyptiacum." So the accounts given of the Assyrian practice of physic mention it as consisting chiefly of incantations or of the prescription of precious stones. But it must be remembered, that this is an account given by those who are unacquainted with a subject, and who very generally are apt to take their own ignorance as the measure, by which they attempt to gauge the knowledge of others. Taking, however, material substances, which are confined to particular soils and climates, we may observe, as in the case of the Egyptians, that many of those produced in Persia were early employed by the Greeks; as, for instance, the several fetid gum-resins which still hold their place in the Materia Medica of the day; also some others, as cuminum, *kumoon;* cannabis, *kinnub;* capparis, *kibbur;* crocus, *koorkum;* carthamus, *koortum;* harmala, *hoormul;* moly, *molee;* susinum, *sosun;* narcissus, *nurgus.* Some of these, again, have travelled southwards, and become known to the Hindoos: assafœtida, for instance, is mentioned in some early Sanscrit works and dictionaries. It is curious in these early accounts of medicine to find so little, if any, notice of the natives of India. This we can account for only by the Hindoos being so far removed, and from their having, on one side the Egyptians, and on the other the Persians, placed between them and the Greeks, from whom we receive our principal accounts. Dr. Whewell, however, in his 'History of the Inductive Sciences,' though inclined to consider that the early efforts of the Greeks in physical speculations, and their philosophy on such subjects, owed nothing to the supposed lore of Egypt and the East, yet makes an exception, "perhaps of the Indians, as the only one of the African or Asiatic nations who ever felt the importunate curiosity with regard to the definite application of the idea of cause and effect to visible phenomena." (p. 32.) Dr. Renouard, the latest author on the history of medicine, and whose work was reviewed in the last Number of this Journal, though admitting the antiquity of the Egyptians, as well as of that of the Chinese, depreciates that of the Hindoos, and referring to some system of medicine, which he calls Vagadasastir, says: "Nulle pensée philosophique n'a présidé, à la distribution de cette encyclopédie méthodique" (p. 45); though at p. 116 he talks of the "antique civilisation Egypto-Indienne," and at p. 132, that "les Asclépiades avaient conservé jusqu'à cette époque les traditions de l'école Egypto-Indienne." But the Asclepiades themselves did not begin to argue, write, or in any way make public their information, until Pythagoras, after his return from the East and his disciples, had set them the example. M. Renouard, however, is in error in ascribing the inferiority of the Hindoos to their belonging to the Mongolian race of mankind: "il paraît que la race Mongole, à laquelle appartiennent les naturels de l'Egypte, des Indes orientales et de la Chine." Now, the Hindoos belong to the Caucasian race as much as any of the nations of Europe, and their language is ranked with the Indo-European, or, as sometimes called, Indo-Germanic languages. The great resemblance between the Greek, Latin, and Sanscrit may be seen in the appendix of an easily accessible work, that is, Dr. Pritchard's valuable 'Physical Researches.'

Though Indian medicine would not appear, as far as our present investigations have proceeded, to have been much known to foreigners, there is little doubt of its practice having been held in high estimation within the limits of their own country. Of this we have indubitable proof in the rock-engraven inscription which has been already alluded to, and which is remarkable, as not only proving the esteem in which medicine was held, but also as showing the high civilization of the governing power. For instead, as is usual with laboured proclamations employed in recording the virtues of a conqueror, or the extent of his conquests, this inscription is a medical edict intended for the benefit of the subjects of

Raja Piyadasi, who has been identified with King Asoka. It directs the establishment throughout his territories of depots of medicines, and the affording of medical aid both to men and animals; and at a period which could not have been long subsequent to the times of Alexander the Great. For it mentions the generals of one of his immediate successors as at that time ruling in some of these provinces, and of which the date is therefore inferred to be 220 years B. C.:

"Everywhere within the conquered provinces of Raja Piyadasi, the beloved of the gods, as well as in the parts occupied by the faithful, such as Chola, Pida, Satiyaputra, and Ketalaputra, even as far as Tambapanni (Ceylon),—and moreover within the dominions of Antiochus the Greek, (of which Antiochus' generals are the rulers)—everywhere the heaven-beloved Raja Piyadasi's double system of medical aid is established;—both medical aid for men, and medical aid for animals; together with medicaments of all sorts, which are suitable for men, and suitable for animals. And wherever there is no (such provision)—in all such places they are to be prepared, and to be planted: both root-drugs and herbs, wheresoever there is not (a provision of them), in all such places they shall be deposited and planted. And in the public highways, wells are to be dug, and trees to be planted, for the accommodation of men and animals."

The records of Indian medicine are not, however, reduced to such scanty notices; indeed, even in the accounts of the ancient philosophy, sciences, and arts of the Hindoos, we find numerous notices of their medical writings. That some of these are still in existence, we have proofs in the original translation and commentary mentioned at the head of this article.

Dr. Wise, in some preliminary remarks, referring to the origin of medicine, and to its subsequent improvement by the Greeks, remarks, that—

"The Grecian philosophers were assisted by the Egyptian sages, who appear to have obtained much of their knowledge from some mysterious nation of the East. Egypt, after having had her institutions destroyed by the sword of the conqueror, became the seat of Grecian learning, which was afterwards transferred to the East, where, under the fostering care of the caliphs of Bagdad, medicine was cultivated with diligence and success. It received still further additions from the East, and thus improved it was conveyed by the Mahomedan conquerors into Spain.

"Among the sacred records of the Hindus there is a system of medicine, prepared at a very early period, that appears to form no part of the medical science, and is not supposed to have enlightened the other nations of the East: a system for which the Hindus claim an antiquity far beyond the period to which the history of the heroic age is supposed to extend." (p. 1.)

Dr. Wise states that he had been induced, from an early period of his residence in Bengal, to examine the Hindoo medical Shastras, and says:

"I translated and compared what I considered the most valuable parts of different manuscripts,—it then occurred to me that the following commentary might be worthy of being published separately, as containing interesting information which had not hitherto been placed before the public. An accomplished scholar* had indeed given an interesting account of Hindu opinions regarding certain diseases: a persevering traveller† had afforded a sketch of certain opinions contained in the Hindu medical Shastras, as translated into the Tibetan language; an anti-

* Professor Wilson, Trans. Med. and Phys. Society of Calcutta, vol. i, and Orient. Magazine, February and March, 1823.

† Csoma de Koros, Journ. Asiatic Soc., Calcutta, January, 1835.

quarian and a distinguished physician* had given some of their peculiar opinions, as found in the medical works of the south of India; and an able lecturer† had combined all this information with important additions of his own; but a comprehensive view of their system of medicine, which it is the intention of the present work to supply, is still wanting to complete our information on the subject."

There can be no doubt of the value and importance of a work like that of Dr. Wise, but the above is hardly a sufficiently full account of the state of our previous knowledge, as Professor Wilson had, in the 'Oriental Magazine,' given us a general idea of the nature of the work of Susruta, and we had some knowledge of their Materia Medica from the investigations of Dr. W. Hunter, of Colebrook, also of Drs. Fleming, Roxburgh, and Ainslie. But it is certainly remarkable that, with the attention which was paid to early Hindoo opinions in philosophy, especially in metaphysics, as well as in science, so little inquiry should have been made, even by medical men, respecting their medicine; so that Dr. Wise be enabled to quote Sir W. Jones as asserting, "that there is no evidence that in any language of Asia there exists one original treatise on medicine considered as science."

In order to show the probability of the Hindoos having made some advance in medicine, Dr. Wise takes a short preliminary notice of their history, chronology, literature, science, and philosophy, as these "are all so combined with each other and interwoven with their theology, as to require elucidation, before the originality of their medical system can be proved, some of their theoretical notions understood, and the probable age determined in which the medical writings were prepared."

The importance of health and the difficulties of the subject no doubt induced the Hindoo sages to accumulate observations, and for the purpose probably of inducing greater reliance to be placed upon them and the advice inculcated, they ascribed both to the assistance derived from their deities. Several of these, indeed, are supposed to have possessed a knowledge of medicine, and who, from taking compassion on the sufferings of the weak and erring creatures on earth, are supposed to have communicated to a few favored mortals the means of preventing and of curing diseases. Hence the assigned origin of some of the medical writings.

1. The *Ayur Veda* is the most ancient system of medicine, and is of the highest antiquity; but fragments only of the MS. are now procurable.

2. The second work, called *Ayugranta,* is said to have been written by Siva, in the *Treta Yuga.*

3. The nature of medicines and diseases is treated of in some of the Puranas, particularly in the Ugni Purán.

4. The names of the following authors, who are said to have flourished under Yudhistira, in the beginning of the *Kali Yug,* are found in the Mahabharatta:

* Dr. Heyne, Tracts on India; and Ainslie, Materia Medica Indica.

† Royle, Essay on the Antiquity of Hindoo Medicine, London, 1837, where the above references are given more in detail, at p. 47.

| Author's name. | Found. | Supposed to be irrecoverable. |
|---|---|---|
| Atrya | Atri Sangita | |
| Ugni Besa / Charaka | Charaka | |
| Bhila | | Bhila Tantra. |
| Jatukarna | | Jatukarna Tantra. |
| Parasara | | Parasara Sangita. |
| Harita | Harita Sangita | |
| Karpari | | Karpari Tantra. |
| Dhanwantari / Sushruta | Sushruta | |

These works are supposed to have been prepared by different munis or sages, on the plan, in a great measure, of the original Ayur Veda. Dr. Wise gives a list of several other works now found in Hindoostan on surgery, medicine, materia medica, &c., and which he has arranged in the probable order in which they were prepared: Systems of surgery—Aupadhanaha and Aurabhra. Systems of medicine—Bhila Tantra, Jatukarna Sangita, Parasara, Harita, Bhagavata, Bhava-prakasa, Todrananda, Chakradatta, Pracharanantabali, Sarangadhara. Systems of Materia Medica—Rajanirghanta, Chakradatta, and Drabyaguna, which is a commentary on the last work. On nosology—Madhaba-Nidana. On pharmacy—Bangaja Ratnabali. On metallic preparations—Rasa Ratnakar, Rasendrachintamani, and Rasendrakalpadruma.

It is evident that a great portion of the interest and value of these Hindoo medical writings must depend upon the period at which they were written. To determine this with certainty we believe to be impossible, from the inattention of the Hindoos to correctness in their chronology. It is fortunate, however, that we can prove the existence of some of these works at a period well known both in the history of the world and in that of medicine; and that is at the establishment of the Arabian school of medicine about A. D. 773, under the patronage of the munificent caliphs of Bagdad. The late Professor Dietz, of the University of Konigsberg, seems to have been the first to prove that the Arabs were acquainted with the Hindoo works on medicine. This is recorded in his 'Analecta Medica,' Leipsiæ, 1833, a work which we have not seen, v. 'Journ. of Education,' vol. viii, p. 176, where it is stated that "Dietz proves that the later Greek physicians were acquainted with the medical works of the Hindoos, and availed themselves of their medicaments; but he more particularly shows that the Arabians were familiar with them, and extolled the healing art as practised by the Indians quite as much as that in use among the Greeks." This information Dietz seems to have derived from the chapter on Indian physicians in the Biography of Ibn Abu Osaibiâh, to which we shall immediately refer. Dr. Royle, in his essay on the 'Antiquity of Hindoo Medicine,' 1837, came to the same conclusion by an independent course of investigation, that is, while employed in identifying the Materia Medica of the East. He found in the Latin translations of Rhases and of Serapion the names of Sarak, Scarak, and Xarek in connexion with the descriptions of drugs the produce of India, and inferred that this could only be the famous Indian writer on medicine, CHARAK or CHARAKA. He further found that the chapter on Leeches by Avicenna commenced with acknowledging his obligation to Indian authorities by saying, "Indi dixerunt," and that the description was word for word that by

Susruta on the same subject, and which had been previously translated by Professor Wilson, and may be seen in Dr. Wise's Commentary at p. 177. Gildmeister, of Bonn, commenced in 1838 a work entitled 'Scriptorum Arabum de Rebus Indicis loci et opuscula inedita.' But he also seems to have derived his information from the above chapter of Ibn Abu Osaibiâh, who lived at the beginning of the 13th century, and died A.D. 1269. This has fortunately been translated by the Rev. W. Cureton, "from a MS. in the "Rich collection" in the British Museum," and is accompanied by some "Remarks on the names which occur in the preceding notices," by Professor H. H. Wilson, in the 'Journal of the Asiatic Soc.' vi, p. 105, 1841. Of the principal names mentioned, Kankah, Shanak, Mankah and Salih, Professor Wilson remarks, that "though they have all the appearance of being Indian, yet that they are names unknown in India. This he attributes to their having a local celebrity only, and, though in great practice and high repute at Bagdad, they may not have been known at Benares or Palibothra; and this was most probably the case, as their knowledge of Arabic and Persian would indicate a long extra-Indian residence. But among the books mentioned are those of Sirak the Indian, and of Sasard, in which are the symptoms of diseases, the manner of treatment, and the medicines to be used for them, and also the book called 'Yedan,' which describes the symptoms of diseases, without the modes of treatment. The first, there can be no doubt, is intended for *Charak*, the name of the oldest Sanscrit physician, and also of his book. The Kitab *Sasard* or *Sasrad* must be intended for the celebrated Indian work, the Susrut, while *Yedan* is, no doubt, intended for *Nidan*, or diagnosis which indicates one division of Indian medicine, on which not only distinct chapters, but separate treatises are written." From all which Professor Wilson concludes that "it is clear that the Charaka, the Susruta, the treatises called Nidan or Diagnosis, and others on poisons, diseases of women, and therapeutics, all familiar to Hindoo medicine, were translated and studied by the Arabs in the days of Harun and Mansur, either from the originals or translations made at a still earlier period into the language of Persia." He concludes that the astronomy and medicine of the Hindoos were cultivated by the Arabs of the eighth century, previous to their studying the works of the Greeks.

Though the Arabian school of medicine, no doubt, did good service in preserving and diffusing a knowledge of medicine for a series of ages, when Europe was so buried in ignorance, that students flocked for instruction to the schools of the Arabs in Spain; it is yet generally acknowledged that they added very little upon the whole to what was previously known. Rhases is generally considered to have been the first author who described smallpox; while Albucasis is acknowledged to have made some improvements in surgery. But the Arabs usually obtain credit for having made considerable additions to our Pharmacy and Materia Medica, as well vegetable as mineral, and to have originated Chemistry. Space will not permit us to enter fully into the question of these additions, or the sources whence they were derived, but we may mention that, in running over the list of drugs described by Rhases and by Avicenna, we perceive no less than 60 or 70 of them which are derived from India, or are mentioned in Sanscrit works; in addition to those which were known to the Greeks. Almost all the above are enumerated in Dr. Hessler's translation of Susruta, or in the Commentary of Dr. Wise. As instances, we may mention—Ambra, Succinum. Anacardium, Semecarpus Anacardium. Bellyrici, Terminalia Bellerica. Baurach kebuli is Birunj kabulee, Embelia Ribes. Kafoor, Camphora officinalis. Cubabeh, Piper Cubeba. Cambil, Rottlera tinctoria, an anthelminthic, like cowhage. Diudar, Pinus Deodara. Deude or Dund, Croton Tiglium. Emblicus, Phyllanthus Emblica. Granum Nil, Ipomœa cærulea, an excellent purgative. Kirm-daneh is seed lac, and Look is common lac. Myrobolani, Terminalia Chebula. Meisee, described as a kind of pulse, is probably *Mash* of the Hindoos, Phaseolus Mungo. Besa or bish, Aconitum ferox. Nux moschata, Nutmeg. "Nux Indica est. neregel," that is, naryul, or Cocus nucifera. Nux methel, Datura methel. Sandalus, Santalum album. Seituragi, Plumbago zeylanica. "Sheel est medicina inda sim. zingiberi," Hedychii species. "Gariophyllus, fructus arboris in insula Indiæ," Caryophyllus aromaticus. Khiar-shumbur, Cassia fistula. Tumr-hindee, Tamarindus indica. Tembul, Piper Betle. Turbit, Ipomœa Turpethum. Kholinjan, Alpinia Galanga. Zedoaria, Curcuma Zedoaria. Zurumbet, Curcuma Zerumbet.

For all these drugs, mentioned often by their Indian names, the Arabs were, no doubt, indebted to the translations of the Indian works, to which we have seen they had access. But some of them may have been previously made known to them by the commerce up the Red Sea, which brought the treasures of the East to the city of Bagdad. There is also reason to believe that translations from Hindoo works were made at periods anterior to the times of Harun-al-Rashid and of Al Mansor, the great patrons of the school of Bagdad. For Rhases, acknowledged to be one of the earliest of the Arabian writers, quotes several others who describe Indian medicines, as Badegora, Mersemai, Persianus, Sindishar, Sindya and Tabri, especially called "Indus." But there is no doubt that there was an earlier school of medicine at Jondisabour, in Persia, and that this will account for the great number of the so called Arabian physicians having been natives of the eastern part of Persia.

That it was the practice to make translations from the Sanscrit into Persian, we learn from the Baron de Sacy, who, in his account of the now well-known Sanscrit origin of the Fables of Pilpay, states that they were first translated in the sixth century, by the physician Barzouyeh, who had made two journeys to India for the purpose of learning Sanscrit, and procuring Indian books as well as medicaments and herbs. As there was constant intercourse between India and Persia, and likewise between Persia and the Greeks, it is possible that the later Greek authors might thus get acquainted with many Indian products; and hence we may account for the appearance in their works of some Indian drugs. Thus Paulus Ægineta, who lived at the end of the sixth and the beginning of the seventh century, and whose work seems to have been translated by Honain at Bagdad, has several compounds which are named Indian, and one of them by a Sanscrit word, 'Trypherum,' used by Susruta and others. Aëtius, who probably lived about the end of the fifth century, mentions "Nuces indicæ," cocoa-nuts; "zador," zedoary; and "galanga" g[illegible]ngal; "santalum" sandal-wood; also the fruit of Semecarpus Anacardium, and an antidote of two kinds of pepper, all of which indicate an acquaintance with, and employment of, Indian products.

Dioscorides, it is well known, describes a considerable number of Indian products, which occur also in the works of his successors down to the time of the Arabs, and which we have every reason to believe to be the same substances for which these authors in many instances give the Indian names, as spikenard, Calamus aromaticus, cinnamon, cassia, malabathrum leaf, or tamala putra, Piper nigrum, and P. longum, and the root of this plant, zingiber, *sans.* shringavera, cardamoms, turmeric, costus, agura or agila wood, converted into aloe wood, bdellium, olibanum. All of these are the produce of India or of neighbouring countries, and most of them we find constantly mentioned in Dr. Wise's Commentary. So *ονυξ*, usually translated Unguis odoratus, seems to be the same substance as the Sanscrit *nakhi,* literally nail, and which is celebrated as a perfume in the Sanscrit Amera Cosha, and is the operculum of one or more species of univalve shells, described by the Greeks as obtained from the *nard-bearing lakes.* Some of the above drugs are mentioned in prescriptions even by Hippocrates, as the two kinds of pepper, cardamoms, ginger, cinnamon, cassia, spikenard, and Calamus aromaticus.

Seeing that so many medicines, the produce of India, and of which the properties must have been investigated by the natives of the country, had at such early periods became sufficiently famous, to have been conveyed to and employed by distant nations, it becomes an interesting question to determine what were the opinions of the Hindoos on medical subjects, and whether their practice was entitled to the esteem, in which it seems to have been held from the rock-engraven inscription.

That the physicians educated according to the then Hindoo system of medicine were esteemed even by strangers, we have the evidence in the proofs quoted by Dr. Wise. "Arrian informs us that, in the expedition of Alexander to India, the Grecian physicians found no remedy against the bites of snakes; but the Indians cured those who happened to fall under that misfortune."

"For this reason, Nearchus tells us, Alexander, having all the most skilful Indians about his person, caused proclamation to be made throughout the camp, that whoever might be bitten by one of these snakes should forthwith repair to the royal pavilion to be cured. These physicians are also said to have made other cures; but as the inhabitants have a very temperate climate, they are not subject to many varieties of disease. However, if any among them feel themselves much indisposed, they apply to their sophists (Brahmans), who, by wonderful and even more than human means, cured whatever will admit of it."

This we conceive is ample testimony to the skill of the Hindoo physicians, according to what was considered such by their Greek invaders 300 years before the Christian era. We are therefore justified in considering that medicine was studied by the Hindoos, and perhaps that some of their works were written at that early but flourishing period of their history. We have seen that the Arabs had access to, and had quoted their two principal works, and that the earliest of the Greeks were acquainted with several of their medicines; hence it has been argued that "this proves the still earlier investigation of their properties, and therefore the cultivation of medicine in the countries where alone these substances are found to grow." Professor Wilson had long since inferred "that, from the Charaka and Susruta being mentioned in the Puranas, the ninth or tenth century is the most modern limit of our conjecture; while the style of the authors, as well as their having become the heroes of fable, indicates a long anterior date." The name of Dhanwantari is found mentioned with those of Charaka and Susruta in poems written in the time of Nala Raja, and in Charaka the names of the Munis or ancient Hindoo sages are alone enumerated, and without the mythology which is so conspicuous a feature of later works. The 'Ayur Veda' is supposed to have been written about the period of Menu's code, or about 900 years B.C., and the Vedas about the 14th century B.C. But these dates will probably become corrected, as progress is made in the present investigations into Eastern antiquities.

From these works we are taught respecting the origin of medicine. First, that the Ayur Veda, or the most ancient medical writings, are considered to form a portion of the fourth or Atharva Veda, conceived to be the work of Brahma, by whom it was communicated to Daksha. By him the two Aswins, or sons of Surya (the sun) were instructed in it; these then became the medical attendants of the gods. A genealogy, as observed by Professor Wilson, that cannot fail recalling to us the two sons of Esculapius, and their descent from Apollo. The Aswins, according to some authorities, instructed Indra, and Indra was the preceptor of DHANWANTARI, but others make CHARAKA prior to him. The pupil of Dhanwantari was SUSRUTA. The works of Charaka and Susruta are the two most ancient which now remain, with parts of the Ayur Veda in the works of commentators; but in Menu we have several notices of subjects connected with medicine.

The Ayur Veda treated of the whole science of medicine under eight different heads. 1. *Salya,* the art of extracting extraneous substances. 2. *Salakya,* the treatment of external organic affections. These two divisions are included in the surgical diseases of modern times. 3. *Kaya Chikitsa* forms what is now included under the head of medicine, as *chikitsa* means the application of the methods of cure to *kaya,* the body in general, and under it are included fevers, dysentery, diabetes, mania, leprosy, &c. 4. *Bhutavidya,* treats of the means of restoring the deranged faculties of the mind, when induced by demoniacal possession. This long continued a subject of attention in Europe, as we may see in the numerous quotations, in the chapter on the Causes of Melancholy in Burton's remarkable work. 5. *Kaumara bhritya,* included the treatment of infants from their birth, and of their diseases, with that of nurses as connected with lactation. 6. *Ajuda tantra,* treated of the administration of antidotes against poisons, whether mineral, vegetable, or animal. 7. *Rasayana tantra,* chemistry, or rather alchemy, as the chief object of the chemical combinations, which are chiefly metallurgic, was to discover the universal medicine, that was to render health permanent and life perpetual. 8, or last part. *Vajiharana tantra* professed to make known the best means of increasing the human race. We cannot avoid noticing the correspondence between this system and that of the Egyptian Encyclopædia of Medicine mentioned at p. 2. At a later period, Charaka relates that the sacred sages or Munis, being grieved at the weakness and sufferings of mankind, assembled in the Himalaya Mountains, resolved on sending one of their number to Indra in heaven, to acquire a knowledge of medicine. Bharadwaja is stated to have returned with a knowledge of *Ayur Veda.*

This was imparted by the sages to numerous pupils for the good of mankind. Several works were written; that of 'Agnibesa' was declared to be the best practical work, but after it was corrected by Charaka it received his name. He therefore became the instructor of practitioners upon earth. This forms the most ancient and the most celebrated Hindoo medical work: it, however, displays less anatomical knowledge, is obscure in the arrangement, though accurate in the description of disease, and is distinguished from more modern works by simplicity in prescription.

The work of Susruta treats—1st, of *Sutra-stana*, or surgery, including, however, observations on climate and different kinds of food as influencing health, with a description of the diseases of the humours, of prognosis, and a notice of different kinds of medicines, in 46 chapters. 2. *Nidana-sthana*, or the description and diagnosis of diseases produced by vitiated humours. The Latin translation of Dr. Hessler only includes these two parts. 3. *Chikitsa-sthana* (translated therapeutics), is treated of in 40 chapters. 5. *Kalpa-sthana*, or toxicology. 6. A supplementary section, including various local diseases. The whole was printed at Calcutta by the Government Educational Committee, in the original Sanscrit.

Dr. Wise has compiled a very useful and interesting commentary, on the knowledge of medicine displayed in the works now procurable in India; and he acknowledges that,

"For arriving at the true meaning of words and expressions, I have had the assistance of able Pundits; of these, I must particularly mention the assistance I have derived from Abhaycharan Tarkapanchánan, now superintendent of the Bengali department of the College of Mohammed Mohsem at Hoogly, and of Madhusudan Gupta, lecturer of anatomy to the Medical College, Calcutta, whose accurate knowledge of the medical Shástras is combined with an extensive knowledge of the sciences of Europe."

Dr. Wise has unfortunately not sufficiently distinguished what he has extracted from ancient Sanscrit medical writings, from that which occurs only in the works of comparatively modern Bengalee commentators. He indeed makes an apology for the opinions of the moderns having in one or two places been stated with those of the ancients. The instances, however, we fear, are more numerous than a reader would be led to expect, from the above form of expression. This we are unfortunately unable to verify, from unacquaintance with the original Sanscrit, as well as with the Bengalee commentaries; but observing several instances in which diseases and remedies are differently named from what we had been accustomed to consider Sanscrit nomenclature, we begged for an explanation of the difficulty from a celebrated Sanscrit scholar. He has been kind enough to point out several instances in which the Bengalee methods of spelling have been substituted for that of the correct and classical Sanscrit, especially in the substitution of *b* for *v*, and *o* for *a*, as is usual in Bengalee works; as, for instance, the name of an author *Bhavaprakasa* is spelt *Baboprakasa*, *Dravya*, substance, is named *Drabya*, elements; *Chyavana* is *Chyabana*; *Daksha* the Prajapati is called *Daka*; and the Rishi *Obree* must be *Atree*. *Amassia* and *Puckasia*, receptacles for undigested and digested food, should be written Am-asaya and *Pák-asaya*. The bile, p. 44, is described as being of a blue colour, the word no doubt is *nila*, signifying black.

Dr. Wise also regrets "to find many errors in the orthography of names."

These errors, he states, "would not have occurred had I not been obliged on account of ill health to leave India, and had I not, on my return to this country (that is, India), been stationed at a distance from Calcutta as the work passed through the press." This must form some apology for the numerous typographical errors which occur, many of which, however, we think, might have been avoided if any one of Dr. Wise's medical brethren had read the proof-sheets. We should not have noticed these errors so prominently, if they did not seriously interfere with the value of the work.

For if Hindoo youths, necessarily but imperfectly acquainted with English, are to study the work, they will inevitably learn incorrect orthography, and be unable to attain further information, from dictionaries, &c. respecting such strangely metamorphosed words; while the European reader cannot avoid losing confidence in the parts with which he is unacquainted, when he finds so much incorrectness in ordinary terms. Taking some of the more remarkable, as, for instance, in the table of bones, at p. 53, we have *Annominata* for Innominata, *Palet* for Palate, *Enciformed* for Ensiform, *Umbiliacus* for Umbilicus, throughout the work. *Ascetics* (p. 184,) for Ascites, which is described as a disease at p. 358, by the name of Asitis, *Amenagogue* (p. 123 &c.) for Emmenagogue, *Raisin* for Resin, *Lucoreah* for Leucorrhœa, *Gravel* for gravid uterus, p. 366, and at p. 381, *seamen* discharged with the menses. All indicating that some of Dr. Wise's native friends have had the chief superintendence of the printing.

While alluding to the orthography of English words, we may at the same time state, that the scientific names are not more correctly given or always accurately applied. The author, moreover, does not sufficiently attend to the period at which Indian products became known to other nations, or to the period when foreign products were introduced into India. Thus he talks of wine being made by Noah, the first year after the debarkation from the ark, which one might suppose was mentioned in some Hindoo work. Sugar-cane is stated to be the sweet cane of Scripture brought from a far country. Now the sweetness here referred to indicates *sweetness* of smell and not of *taste*, and is generally supposed to refer to the Calamus aromaticus of the ancients. Oranges, lemons, citrons, &c., are stated, at p. 102, to have been well known to the Greeks and Romans. There is no proof that any of them were known except the citron. At pp. 416 and 417 we have the pine apple and custard apple mentioned, and at p. 274, potatoes recommended as an article of diet. All these are well known to be American products, and of comparative modern introduction. On the contrary, we have European products mentioned as if occurring in these Hindoo works, and all with Sanscrit names applied to them, but without any proof of their having been known at early times. Thus various names, as tikta, caturohini, ushira, katuka, are translated hellebore, which is itself spelt in various ways. So *Vacha* or *Bacha* is in some places translated orris root, and, at p. 428, *Iris germanica*, but at p. 309, correctly *Acorus calamus;* it is known everywhere in India by the name of *buch*. But we cannot conclude these observations without adverting to Dr. Wise's plan of giving the Sanscrit name along with the English or scientific appellation, as a very excellent one, and worthy of adoption in all translations of such works.

From these blemishes in Dr. Wise's work, we proceed to notice the great mass of curious information which has been incorporated in his Commentary, and which would be much more valuable for tracing the history of medicine, if he had in all cases, as he has done in many, given the name of the original Sanscrit or Hindoo author, whence he derived his information. As in the early ages of Grecian medicine, so in that of the Hindoos, medicine was studied by philosophers who treated of the origin of all things, as well as of the body in particular, its diseases and treatment. Thus—

"At an early period the Hindoo philosophers reduced the material world to five elementary principles and primary qualities, by the agency of which they explained the appearance, composition, and condition of the world, and the structure and functions of the body." (Wise, p. 30.)

The five elementary principles they considered to be earth, water, air, fire and ether, which, as stated by Dr. Wise (p. 31), "was supposed to be separated from the others, and to possess the property of sound and form;" to be "altogether undistinguishable by our senses, and is only made known to us through the evidence of our understanding." But a high Sanscrit authority informs us that the fifth element is properly considered to be equivalent to space, and to be the element that keeps the molecules of bodies from cohesion. We proceed with a few more extracts from this part of the work:

"These elements are all nourishing to the body, and are contained in different proportions in every part of food, so that after digestion each element, by an inherent property, joins with that which already forms a part of the fabric of the body........There being both an active or warm, and a passive or cold principle, which are increased and strengthened by the rays of the sun and moon."

Each of the fluids is also supposed to be influenced by one of the seven planets, which regulates its condition.

All living bodies, among which vegetables are included, are supposed to be composed of the above elements, "with the element-producing action or life superadded. Living bodies are produced from vapour, vegetation, incubation, and parturition, as insects, plants, fishes, reptiles, birds, and animals."

The essential or elementary parts when mixed, form vital bodies, which are divided into two classes. One stationary, the other moveable. This class is divided into four groups: 1, such as are produced from the womb; 2, from eggs; 3, from the warmth of the earth; and 4, from what is written "udjbidgo, such as break their habitation," but we are informed the word is derived from *udbhad*, sprouting, and *ja*, born from, and should be *udbhijja*. Plants are likewise divided into four groups: 1. Trees with fruit but without flowers. 2. Trees with both flowers and fruit. 3. Creepers. 4. Annuals.

"The same elements and quantities, by their combination and action, constitute the human body, which is governed by an independent principle, or soul, which acts through the medium of the members, and is an emanation from the great soul of the world, into which, after certain purifications, it is again absorbed. As long as the soul remains in connexion with the body, the diseases with which it is afflicted may be removed, and it is proper that, during all this time, remedies should be employed for the purpose." (Wise, p. 32.)

From the above few extracts, we perceive that the Hindoos were careful and attentive observers, and that in their general views, however incorrect, may be perceived the germs of many theories which have prevailed at different times. Without adverting to their mathematical knowledge or their systems of philosophy, we may judge from their observations of nature that they were quite competent to make advances in medicine, which requires careful observation, correct induction, and well-considered generalization. We shall proceed to give specimens of their information and opinions respecting different departments of medicine, and notice how far they were acquainted with the accessory sciences, such as chemistry. In comparing these opinions with those of other ancient nations, we must not forget that even among the early Greeks there was no knowledge of anatomy, except that of the bones; that their physiology was fanciful, but that they had much valuable information on the subjects of climate, of hygiene, and of diet. The same we find to be the case with the early Hindoos. We shall first, however, give their notions on the character and duties of a physician.

The Hindoo physicians now form the caste of Vaidhyas (or those who understand the Vidya or Ayur Veda). It is stated that "Brahmans learn the medical Shastras for their advantage, Khetryas (or the military class), for the benefit of their health, and Vaidhyas for their subsistence." A good teacher is like rain falling upon the germinating seed, and should possess the following qualifications: a perfect knowledge of all Shastras, joined to extensive practical knowledge and skill, &c. The medical student should be the son of a respectable and ancient family, who is either the son of a practitioner, or of one who respects the medical profession," &c.

"There are four circumstances required in the cure of a disease:—a physician; a disease that is known; a reasonable patient; and medicines, instruments, and attendants. The acquirements of a good physician are described as consisting, first, in a knowledge of books, without which he will be confused, like a soldier afraid in the time of action; on the other hand, a want of practical knowledge will impede his advancement, and his senses will be bewildered when called on to treat acute diseases." (p. 16.)

"When such a Vaidya is spoken to by a patient in a peevish and hasty manner, he will remain calm, mild, and courageous, and cherish a cheerful hope of being able to save the sufferer's life." (p. 18.)

Pretenders to a knowledge of medicine were known in those days as at the present time.

"They are sometimes allowed to practise by the neglect of the Rajah, and they may be known by their vanity and ill-will towards the good physician. Such persons avoid the society of learned persons as they would a jungle." (p. 19.)

In visiting a patient, the physician is directed "to observe the state of the planets, the time of day, and the good and bad omens." He should then proceed to "ask questions of the attendant regarding the disease, what things he has eaten, and what he has done to produce or to influence the disease." He "should then mark the signs of longevity in his patient, and proceed next to mark the nature of the disease, and which of the humours is diseased, and how they can be cured. He should examine the symptoms of the disease with his eyes, and consider the probable result of the disease by his judgment. The symptoms enumerated in the Shastras should be observed, more especially the state of pulse, of the tongue, as to moisture and dryness, the condition of the bowels, urine, and sleep; his general feeling, more especially the state of the nose, head, hands, feet, and abdomen. By the touch is distinguished the feverish heat or coldness of the surface, the dryness, moisture, &c. By the *hearing*, the passage of air in deep-seated abscesses, wounds, and in the intestines by coughing, &c." By speech, the practitioner learns the time of invasion and progress of the disease, &c. (p. 24.)

"As long as life remains in the root of the throat, and the senses remain perfect the physician may give medicines, as the persons, under such circumstances, may be cured." The physician is, however, to be careful of his own reputation, as well as of the credit of his profession.

"It is proper, however, that much caution be used in the employment of medicine in fatal diseases, as a physician may alleviate pain but cannot give life; and by administering medicine in such cases, without previously stating the danger the patient is in to his relatives, he will bring discredit on himself and on his profession. Taking such precautions, the practitioner may give medicines even when the patient is senseless, without any pulse and any breathing."

Some directions are then given about the collection and preparation of medicines; and the chapter concludes with the recompence of the physician.

"The messenger should always offer a present to the physician. Before the patient takes the medicine, the god of physic is to be worshipped in the person of his deputy, the physician, who must be well paid for his services.

"When a physician has cured a disease he is entitled to the usual gifts for the performance of a good action. These will vary with the rank and condition of the patient. Money will be the recompence bestowed by the rich; friendship, reputation, increase of virtue, prayers, and gratitude will be that of the poor. When a Guru, a Brahman, or a Dandi (fakir), a relative, a humble and good friend, or one without relatives, consults a physician, he must not accept of any pecuniary recompence. His reward in such cases will be an increase of knowledge, and the gratification of his desires in having an opportunity of performing a good action." (p. 29.)

Chemistry is usually supposed to have originated with the Arabs, but without referring to alchemy, there can be no doubt that this science originated with, or was known to the Hindoos previous to the time of the Arabs; and we will take Le Clerc's distinction, "qu'il faut bien distinguer entre la chimie qui enseigne la mélioration ou la transmutation des métaux, &c., et celle qui n'a pour but que la préparation des médicamens, et dont l'objet est la santé."

Even in this view we find many chemical preparations were prepared; thus, to make caustics (p. 181), the ashes of different plants, which we know must have yielded carbonate of potash, are directed to be dissolved in water, and "some shell-lime is then to be mixed with them." The process is the same as that for making solution of potash, according to the Pharmacopœia. Common salt, carbonate of soda, borax, nitre, and sal-ammoniac were well known; to the last, lime-water is directed to be added, in a bag (probably a leathern water-bag), to prepare, what must have been, a solution of ammonia. Iron and tin are stated (Wise, p. 117) to be the only metals which were prescribed internally by the more ancient Hindoo physicians; but in the translation of Susruta, p. 95, we find stannum, plumbum, cuprum, argentum, lapis magnes, aurum, et rubigo ferri. "At a later period, mercury, gold, silver, copper, lead, and zinc were introduced and several preparations made from them." Metals are, however, usually prescribed only in the form of preparations ; the sulphuret of antimony, as well as that of arsenic and arsenious acid seem to have been used at very early periods ; as in the translation of Susruta, pp. 85 and 95. We have also the three kinds of vitriol, or the sulphates of iron, of copper, and of zinc, prescribed in different diseases ; and directions are given for the making of various preparations, especially of mercury. But here we have again to notice carelessness in nomenclature, with errors in the substances named. Thus cinnabar, or red sulphuret of mercury, is directed to be made with sulphur and *blacklead.* The sulphuret is sometimes called sulphate, but most frequently sulphurate of antimony. Kusees is translated sulphate of iron at p .166, and sulphate of zinc at p. 302. Accurate translations of some of the earliest formulæ for making these chemical preparations, would be extremely interesting as enabling us better to trace the history of chemistry.

The Hindoo Pharmacy may now be noticed, and appears to be sufficiently complete, as weights and measures are attended to, and medicines are apportioned according to age, and prescribed in the form of powders, pills, fresh juices, pastes, decoctions, and infusions ; extracts, roasted medicines, electuaries, oils, and spirituous mixtures. The menstrua in which medicines are to be given are water, honey, sugar, &c., or such substances as speedily act on the body.

" The time for administering medicine is important, some requiring to be given before, others during, and a third after eating. The general opinion is, that medicines should be taken on an empty stomach, as it is then soon digested, and like a drop of oil let fall upon water, is taken into the system and diffused quickly over it.

" Medicines given in too small doses will be like throwing a little water upon a large fire, that rather increases than diminishes it. In like manner, too large doses of medicine will increase the disease, and will be liable to produce other diseases."

Having seen that not only the Arabs but also the Greeks had been indebted for many of their drugs to India, we should naturally expect to find these noticed in their medical works. In this we shall not be disappointed. Indeed we find nearly all those which we have already noticed as having been known to the Greeks and Arabs. There are also many others which still continue to be used by native practitioners in India, and which are possessed of useful and active properties. Among all these the only exotic drug which we observe is assafœtida. Dr. Wise states, that Charaka arranges simple medicines under forty-five heads. Many of the groups are intended only to relieve particular symptoms ; others as sedatives, stimulants, tonics, emetics, purgatives, &c., are such as we still continue to employ. Susruta divides medicines into two classes : those which evacuate bad humours from the body, as purgatives and emetics ; and those which diminish the exalted action of the humours, and restore them to the healthy state. But he also arranges them under thirty-seven different heads, which are given in the translation of Dr. Hessler. Many of these divisions, as those of Charaka, are intended for the treatment of particular diseases or of symptoms only. Subsequently, (chap. 43,) he treats of emetics, and among the principal of these enumerates Vangueria spinosa, which is interesting as belonging to the same natural family as ipecacuanha; Asclepias geminata, azadirachta, the Indian species of mustard, bitter cucurbitaceæ, fossil salt, and others. In the following chapter, (xliv.) he treats of purgatives. Among these we find danti (not translated by Dr. Hessler) or Croton polyandrum, a substitute for Croton Tiglium, Ricinus communis, Cassia fistula, purgative cucurbitaceæ, the root of Convolvulus Turpethum, and the seeds of Ipomœa cærulea, both excellent purgatives, myrobolans, the juice of Euphorbia antiquorum, others less known, with some milder purgatives, as sugar, fruits, &c. These are usually combined with some warm aromatics, such as pepper, ginger, and the Costus so well known to the ancients. This Dr. Wise translates Costus speciosus, an inert substance, and Dr. Hessler Costus arabicus, but which being still in use in India, and known by the name of *koot* and *koosta*, was proved by Dr. Royle to be this costus, and traced by Dr. Falconer into Cashmere, where he found it growing on the mountains, and which, as belonging to a new genus, he named Aucklandia Costus verus. Errhines were much employed, as exciting a discharge, by which the head was supposed to be cleared from the presence of bad humours. Dr. Wise says, "few narcotics seem to have been known, except the species of datura and the resin of hemp, or Cannabis indica." But they employed Aconitum ferox, bish; Kakola is Cocculus indicus, and Kaephul may have been Strychnos Nux Vomica. Among the anthelminthics we observe viranga or biranga frequently mentioned, which Dr. Wise does not translate, but which Dr. Hessler correctly renders Embelia Ribes. Of this the small berries, something resembling those of black pepper, are still employed as anthelminthics in different parts of India. We also find in Susruta, roots and seeds grouped together in fours and fives, e. g. the five smaller and the five great roots, as continued to be so long the case in Europe with many roots and seeds. Since we cannot devote more space to this subject, we may conclude with the observations which terminate the chapter on drugs. "When administered by an ignorant person, medicine is compared to poison, is like the knife, fire, or lightning; but when administered with the necessary knowlege, medicine is like amrita, or the water of immortality." (Wise, p. 157.)

As none of the nations of antiquity were well acquainted with anatomy, it is not to be expected that the Hindoos had acquired any great knowledge of the structure of the human body. They did not, however, entirely neglect it, and their mode of dissection is certainly curious and original, if not the most satisfactory. Charaka states that a practitioner should know all the parts of the body, both external and internal, and their relative positions with regard to each other. Susruta states, in addition, that it is by combining a knowledge of books, with practical dissection, that he will attain a knowledge of his profession, and possess an intimate acquaintance with the diseases to which the body is liable, and perform surgical operations, so as to avoid the vital parts.

"The body which is to be examined by dissection, should be that of a person who had neither been destroyed by poison, nor had died of a long disease, should not have been very old, and all the members perfect. When a proper body for the purpose has been selected, the dejections are to be removed, the body washed and placed in a framework of wood, properly secured by means of grass, hemp, or the like. The body is then to be placed in still water, in a situation in which it will not be destroyed by birds, fishes, or animals. It is to remain for some days in the water, when it will have become putrid. It is then to be removed to a convenient situation, and with a brush made of reeds, hair, or bamboo-work, the body is to be rubbed, so as by degrees to exhibit the skin, flesh, &c. which are each in their turn to be observed, before being removed." (Wise, p. 69.)

The body is said to consist of humours, that is, of air, bile, and phlegm, (which are described as the three pillars or supports of the system,) and of the seven essential parts, consisting of the hard and soft parts and fluids of the body, as the chyle, blood, flesh, fat, bone, marrow, and semen. But osteology, as with the most ancient Greek physicians, is the only part that seems to have been correctly ascertained. The number of bones are stated by Charaka, including some cartilages, to be 306, while Susruta states them to be 300 in number. The different forms of bones and the several kinds of joints are enumerated. Ligaments are described as binding together the bones, as strips of ratan bind together the pieces of a boat. "Muscles cover, strengthen, and retain in their place vessels, tendons, bones, and joints." Much absurdity is related respecting the vessels, which are said all to originate from the navel. They "are like decayed leaves, in which the interstices have been removed" but "nourish the body as a garden is irrigated by a small brook." Besides these, canals, fascia, organs, receptacles, and the orifices of the body are noticed. The vital parts are named and described by Susruta, and the necessity of avoiding them in operations pointed out. Thus, if the vital parts in the palm of the hand are wounded, the arm is to be amputated, to save the individual's life. So if the urinary bladder be wounded, the person will soon die, except after the extraction of the stone. If the bone of the head or breast be broken, it is to be raised by the assistance of instruments. Some wounds in vital parts prove fatal on the withdrawal of the instrument inflicting them, others, if the fatal termination is at first prevented, will prove fatal after some days, with much suffering and weakness. Some of these consequences must no doubt have been observed from the effects of sword and spear wounds; but the whole of the information under this head indicates much greater attention to the subject of anatomy, than could have been supposed possible, by those best accquainted with the Hindoos.

Surgery, which is named Sutra sthana in the Ayur Veda (Wise, p. 8) and Shala at p. 157 (and it forms the first chapter of Susruta), seems to have been first practised by the Hindoos as it was also by the Greeks. Dhanwantari, having asked his pupils on what he should first lecture, they replied on surgery, because there were no diseases among the gods; wounds being the first injuries which required treatment. Besides, "the practice of surgery is more respected as affording immediate relief, and is connected with the practice of medicine; although the latter has no connexion with surgery," (p. 8); but at p. 158 Dhanwantari declares "that surgery cannot be practised with success unless the practitioner is familiar with the practice of medicine, of which it is only a branch." In the book of Susruta, in 46 chapters, many things are included which we should now place in other departments, as the general duties of teachers and practitioners, the diseases of the humours, of things useful or hurtful to the different humours, the difference of climates, of winds, the several classes of medicines, and the different articles of diet. But in this book are also included the different kinds of bandages and the selection of instruments, description of some surgical diseases, the removal of extraneous substances, the restoration of defective ears and noses, the different stages of inflammation, the treatment of wounds and ulcers. Inflammation is described as of two kinds: the one produced by external injuries, and the other by derangements of the air, bile, phlegm, and blood, or their combinations. The different methods of performing venesection are described, and the parts of the body whence blood should be taken, according to different diseases, with a notice of the variety of modes in which the operation may be improperly performed. The actual and potential cautery are fully described, and seem to have been frequently employed. It is observed that, "as cutting, fire, &c. give pain, rajahs, rich people, children, or old people, and fearful and weak people, when they require to lose blood, may have leeches in preference to venesection." The several kinds of leeches are then described, and the mode of cupping with a horn out of which the air is sucked out, or with a hollow gourd, of which the air is exhausted by burning something in it.

In the 9th chapter of Susruta, the student taught science by books, is next to be instructed in the practical use of instruments, &c., for "without practical skill theoretical knowledge is of no use," or, as Dr. Hessler translates it, "Perquam edoctus enim, deficiente exercitatione, in operationibus inexercitus est." (p. 18.) The different surgical operations are to be shown to the student upon wax spread out on a board, on gourds and other soft fruits; tapping and puncturing on a leather bag of water or of soft mud; scarifications and bleeding may be practised upon the fresh hides of animals, from which the hair has been removed, or upon dead bodies, and by puncturing or lancing the hollow stalks of water lilies, or the vessels of dead animals. The removal of substances from cavities by removing the large seeds of the jack (Artocarpus integrifolia) or bel (Ægle marmelos) fruit. The extraction of teeth is to be practised upon dead bodies and animals. The mode of making noses, ears, &c., must be practised upon dead animals.

These practices are mentioned and adduced as instances of how different is not only modern from the ancient surgery of India, but as proving that the prejudices and modes of thinking respecting the dead bodies of men or animals must have been very different from anything we now know of the Hindoos. Surgical operations are described as being of 8 kinds.—1, incisions; 2, opening of abscesses; 3, scarifying parts; 4, puncturing, as in hydrocele and dropsy; 5, probing to ascertain presence of foreign substances; 6, operations of extraction, as of the stone, the fœtus ex utero, the teeth, &c.; 7, removing fluids, as pus, blood, &c.; 8, sewing parts together (Wise p. 170). But several operations which the ancient Hindoos seem to have performed are not enumerated among these. Hemorrhage is to be arrested by the use of astringents, cold, ice, caustics, and by the actual cautery. When an extremity is separated, immediately pour boiling oil on the surface, then apply a cap-formed bandage and remedies to heal the wound, (Wise, p. 188). This book concludes with the treatment of fractures and dislocations. The whole shows that at an early period the Hindoos practised many operations, and had paid attention to several of the objects of surgical science.

The first two books only of Susruta having as yet been translated by Dr. Hessler, we are confined almost entirely to Dr. Wise's Commentary for the Hindoo practice of physic. The second book, however, of Susruta, entitled Nidan sthana, usually considered to treat only of diagnosis, is entitled Pathologia, by Dr. Hessler; and we have in the sixteen chapters composing this book the opinions of Susruta on the pathology of the humours and of some diseases. Dr. Wise commences his chapter with the Hindoo opinions on disease, which are those of the humoral pathology, and says (p. 193):

"Nature, which exhibits the highest degree of order in her operations, is liable to occasional irregularities from the impurities and the imperfect manner in which the elements and qualities are mixed together. In like manner, the harmony of the humours of the body is liable to derangement. At one time the disease is owing to an increase of one of the principal humours, at another to its diminution, with regard to the other humours. They thus explained the occurrence and varieties of disease. The soul (jivita) of the body, like the great soul of the world, tended to retard these derangements, or restore such irregularities. When disorder has been introduced, the soul (vis medicatrix naturæ) tends to reduce the humours that are increased, and to augment those which are diminished. In like manner, certain medicines have peculiar effects in producing these changes, and thus assist the soul in her salutary influences.

"Disease is therefore the pain (dukkha) of the soul, caused by the derangement of the humours." (p. 194.)

Diseases were, however, unfortunately arranged, it is said, "according to their prominent symptoms, and not according to the peculiarity of the symptoms and their combinations." A modification of the classification of the Ayur Veda is said to be usually followed in Hindoo medical works.

Charaka divides diseases into three classes, mental, bodily, and accidental, which he supposed were situated in the semen, chyle, or blood. The general causes of disease are also three, proceeding from matter, or objects of sense; the second from improper exercise; and the third from the seasons. "There are also three sorts of medicines: one sort that cleanses the body, when taken internally, as emetics, purgatives, &c.; another sort purifies the external body, when applied externally, as oils, bathing, diaphoretics; and the third kind is the use of knives or instruments, fire and escharotics."

Charaka states also, that there are three objects of inquiry in the world: the first and chief inquiry being the means of preserving life; the second, the means of acquiring wealth; and, lastly, the means of obtaining beatitude in the next world.

The descriptions of disease, Dr. Wise states, are generally distinct and satisfactory; but sometimes it is difficult to distinguish the disease intended, because an accidental combination of a few symptoms seems to be sometimes mistaken for a distinct disease, and in other cases these are placed as varieties of other diseases.

"The description of a disease is usually commenced with an enumeration of the supposed causes, situation, and humours deranged, as indicated by the symptoms and the varieties produced by the humours affected" (p. 198.)

Before proceeding to a description of particular diseases, detailed indications are given of the morbid changes of the humours which enter into all diseases. (p 199.)

Under the head of diagnosis, it is stated that the nature of a disease is to be ascertained by the five senses and asking questions, as 1st, by hearing he will distinguish the state of the lungs, by the peculiar noise of the breathing, &c.

"Nothing is said in Charaka and Susruta respecting the pulse." (Wise, p. 203.)

Prognosis was a department of medicine early paid great attention to, as we see in the works of Hippocrates, and so it appears to have been by the ancient Hindoo physicians:

"As a flower prognosticates the future fruit, smoke the severity of fire, and clouds the near approach and the severity of the coming shower, so certain symptoms prognosticate the favorable or fatal result of a disease. These signs are, however, but slightly apparent to the general eye, and can only be detected by the eye of the experienced physician." (p. 205.)

Several pages are devoted to this subject, but we cannot spare space for extracts. Either Dr. Wise or his Hindoo friend concludes the chapter with observing, that some of these remarks are just, and some are too much neglected by European physicians: "and hence the error which they sometimes fall into." (p. 212.)

With regard to the general treatment of disease, it is stated, that the indications are—"to promote the just balance of the elements and humours, by a judicious choice of aliment, and by such means as assist the vital principle in the completion of the assimilation," attention being first paid to diet: the object in view is to be effected by emetics, purgatives, or bloodletting.

"But these humours are not to be dislodged indiscriminately, but at certain seasons and diurnal periods of the disease. Health was thus supposed to be promoted by the exhibition of an emetic once a fortnight, a drastic purgative once a month, and bloodletting twice a year, at the change of the seasons. The vital principle was supposed to give warning when the corrupted humours were ripe for being evacuated, and the physician was directed to observe carefully, so as to be able to assist and not disturb the spontaneous efforts of Nature. The seasons at which she exhibited these beneficial influences were supposed to be determined by the known cause of the disease, which led to the belief of the definite course and the mystical powers of numbers, by which Nature may be invariably observed to arrive at certain determinate results, which were supposed to be regulated by an arithmetical progression. This led to the belief of the maturation of the diseased humours, and of the existence of a period in which the perfect state of mixture takes place. These were called *critical* days.......These days were recognized by the Egyptian priests, as related by Pythagoras and others. Among the Hindoos the humoral pathology appears to have originated without any assistance from other nations, and became as generally believed, and arrived to the same consequences as it was in Europe." (Wise, p. 212.)

"Another equally plausible opinion was, that all diseases divide themselves into two great classes, of sthenic or (and) asthenic disease. The one being an increase and the other a diminution of excitement, between the extremities of which health was supposed to be placed. This appears to have been an early opinion among the Hindoos, is now generally believed over all the Asiatic nations, and has led them to the division of remedies into stimulating and cooling, which were employed according to the nature of the disease." (p. 213.)

After an account of the diseases of the humours, Dr. Wise proceeds to give the opinions of the Hindoos on fever, and it is said:

"Fevers are first considered, because it is said that man is born and dies in fever; because it affects the whole body, the organs of the senses, and the mind; and is so severe that only men and gods can survive, and by which various other diseases are produced." (p. 219.)

The causes, symptoms, and the varieties of fever are then described, with detailed instructions of the treatment, and with much of what must have been necessarily the result of careful observation, we have incorrect theory and undue attention to numbers. With regard to critical days it is said:

"The 7th, 10th, 12th days, are always the days on which the fever is severe, or from these periods the symptoms diminish in severity.

"In other books it is stated that the critical days are the 7th, 9th, 11th, 14th, 18th, 22d, from which period it is diminished or increased. Those who live to the 22d day generally recover."

Smallpox, of which, however, measles and waterpox are made varieties, is adduced by Dr. Wise as a disease existing for a long period in one country without penetrating into another. It seems to have been long known to the Chinese and Hindoos: he concludes that it was probably conveyed westward by the Persian conquerors of Hindoostan. Rhases is supposed to have been the first to describe it; but, though Dr. Wise does not adduce his authorities, it is probable that accounts of the smallpox are contained in the works to which the Arabs had access.

As we might expect, the subject of leprosy is rather fully treated of, but under this head some other cutaneous diseases are described. "Though it commences first in the skin, it gradually extends deeper and deeper, like the small shoots of the banian trees (Ficus indica), which at first confined to the surface, advance deeper and deeper, until they extend over the whole body."

Among the diseases affecting the mind, we have a chapter on Devil madness. Among those of the head; it is said of hemiplegia, that it "is very obstinate, and most distressing, so that a thousand physicians cannot cure it." The diseases of the ear are said to be twenty-eight in number, and as cutting off ears was a frequent method of punishment, Susruta recommends the surgeon to prepare a new ear, by "removing the skin from the neighbouring part, leaving a connexion to keep up the vitality." Diseases of the eye seem to have attracted much attention, and the operation for cataract is described, but it is not stated from what work this is taken. (Wise, p. 303.)

In the treatment of asthma, we observe no notice of datura-smoking, which is now so generally practised in different parts of India.

Consumption, defined as a rupture or ulcer of the respiratory organs, is entitled the prince of diseases, from its frequent and fatal nature; but we suspect that other diseases, as land scurvy, &c., are sometimes confounded with consumption, which is rather a complaint of the northern, than of the southern parts of India. Urinary diseases seem also to have obtained considerable attention; and we may observe that diabetes mellitus appears to have been known to the Hindoos long before it had been noticed by others. Of urinary calculi it is said, the disease is dangerous like poison or thunder. "When recent, it may be cured by medicine, but when of long standing, an operation is required." The operation is then described, but the authors in which the earliest accounts occur are not mentioned. Among the diseases of the organs of generation, syphilis is treated of by Dr. Wise; but he states that it is not mentioned in old Sanscrit works, but was introduced into Hindoostan by way of Persia, and also by the Portuguese into the southern parts of India, and in comparatively recent times.

Towards the conclusion of the work there is a chapter on "Poisons and their Antidotes." The poisons are usually arranged into two classes: the first, consisting of vegetable and mineral poisons, are named *Stábarch,* and the second, animal poisons, *Jargamah.* The first class is, however, afterwards divided into mineral and into vegetable poisons; but we have considerable doubts respecting the Sanscrit names being correctly translated. Among the animal poisons, serpents, poisonous rats, the bites of mad dogs, and the poisonous bites of insects are enumerated. We are sorry to observe here the same inattention to correctness in translating the names of antidotes as we observed in the case of medicines: thus *Kusta,* which is elsewhere translated *Costus* (p. 403), is called Strychnos Nux Vomica, and mentioned as an antidote to be given internally, and also applied externally, after the parts poisoned have been scarified and burned.

Having taken a general view of the contents of the celebrated work of Susruta, as far as the Latin translation has as yet been published; and of Dr. Wise's Commentary more briefly than we should have done if the authorities had more generally been given; because many of the opinions and statements derive their chief value, from being found in medical works which can be proved to have been in existence prior to the time of the Arabs. Any later opinions may have been derived, though this is not very probable, through the medium of the Persian translations of Arabic works, which are used everywhere in India by the Mahommedan practitioners of medicine. But the parts of the Ayur Veda which still remain, as well as the works of Charaka and of Susruta, prove that the Hindoos had a system of medicine, containing much of original observation and of opinion, before the Arabs had paid any attention to literature or science. Among the opinions and reasons, however crude, of these early medical writers, we cannot but observe a remarkable coincidence in many points, to opinions which were entertained in early times both in Egypt and in Greece. It still remains an interesting subject of inquiry to ascertain the causes of this general resemblance; whether all were indebted to some common source, as to the early civilized and powerful empires of Assyria, Babylon, or Persia; or whether medicine was cultivated first by the Egyptians, or by the Hindoos, and from the one spread to the other: the Greeks deriving their information only from those who were placed nearest them. At all events, we cannot but admit, after the evidence which has been adduced, that, however degenerated may be the natives of India at the present day, there can be no doubt, that at an early period they paid attention to, and had made advances in different departments of medicine. We must also express a wish that these early works should be carefully translated, and the synonymous scientific terms correctly applied, so that we might be able to weigh more accurately the evidences of originality, or trace the mass of curious information to some early source. We hope that the Sydenham Society may be induced to undertake the translation of these early works, accompanied with medical and scientific commentaries, as has been so ably done by Dr. Adams with the translation of Paulus Ægineta.

In conclusion, we could not but have wished that the medical talent displayed by this early civilized nation should have opportunities of cultivation in the present day, but we call to mind, that we have in a former volume, reviewed a work embracing the Reports from the dispensaries in Bengal, where the Practice of the Native assistant surgeons, educated in the Medical College established by the East India Company in Calcutta, is given in detail and in their own words. Colonel Sykes read a paper before the British Association at Southampton, and now published in the Journal of the Statistical Society, March, 1846, on the further progress of these Europeanly educated native practitioners of medicine, where further proofs are given of continued improvement and of extended usefulness. As similar institutions have been established in Madras and in Bombay, there cannot fail to be raised a class of well-educated practitioners, who will carry the benefits of modern science and of greatly improved medical practice, into every part of the widely-spread dominions of the British East India Company.

---

